# Impact of Adding Immune Checkpoint Inhibitors to Neoadjuvant Chemotherapy on pCR and Tumor Downstaging in Resectable Gastric Cancer: A Meta-Analysis

**DOI:** 10.3390/cancers18142270

**Published:** 2026-07-15

**Authors:** Mariam Grazia Polito, Luisana Sisca, Davide Caruso, Antonella Cosimati, Federica Lo Prinzi, Carla Adriana Ramirez Reinaga, Etien Leka, Daniele Santini, Gian Paolo Spinelli

**Affiliations:** 1 UOC Oncologia Territoriale, ASL Latina, CDS Aprilia, La Sapienza Università Di Roma Polo Pontino, 04100 Latina, Italy; 2 Department of Medical Oncology, Fondazione Policlinico Universitario Campus Bio-Medico, 00128 Rome, Italy; 3 Department of Translational Research, Institut Curie, 75005 Paris, France; 4 Oncologia, Presidio Ospedaliero Umberto I, 00161 Siracusa, Italy; 5 Department of Medical and Surgical Science and Biotechnology, Università “La Sapienza”, 04100 Rome, Italy

**Keywords:** immune checkpoint inhibitors (ICIs), gastric cancer, perioperative chemo-immunotherapy, pathological complete response

## Abstract

**Background/Objectives**: Despite standard perioperative chemotherapy, recurrence remains frequent in resectable gastric cancer (GC). We investigated the clinical impact of integrating immunotherapy into the neoadjuvant component of perioperative treatment, particularly on pathological complete response (pCR), in this setting. **Methods**: We conducted a systematic review and meta-analysis of phase II–III trials and real-world studies evaluating immune checkpoint inhibitors (ICIs) added to perioperative chemotherapy versus chemotherapy alone in resectable GC. Two independent reviewers screened PubMed and Scopus; review articles and single-arm studies were excluded. Pooled odds ratios (ORs) were estimated using random-effects models, and heterogeneity was assessed with the I^2^ statistic. Overall, six studies including 2890 patients were analyzed. **Results**: The addition of ICIs significantly enhanced tumor regression, increasing ypT0–T2 rates (OR 1.61, 95% CI 1.11–2.33; *p* = 0.012) and reducing ypT3–T4 residual disease (OR 0.62, 95% CI 0.44–0.87; *p* = 0.0051). A favorable trend toward nodal downstaging was observed (ypN0–1 OR 1.57; ypN2–3 OR 0.60), although these differences did not reach statistical significance. Notably, pathological complete response (pCR) rates more than tripled with chemo-immunotherapy (OR 3.39, 95% CI 2.21–5.20; I^2^ = 38.9%; *p* < 0.0001). The magnitude of benefit was particularly striking in PD-L1-positive tumors (OR 3.26; *p* < 0.0001). pCR improvement was strongest with PD-1 inhibitors (OR 4.10; *p* < 0.0001), while remaining significant with PD-L1 inhibitors (OR 2.62; *p* < 0.0001). Treatment benefit was consistent across age groups (<65 and ≥65 years) and performance status (ECOG PS 0–1). R0 resection rates were not significantly different. **Conclusions**: Overall, the addition of ICIs to perioperative chemotherapy substantially increases pathological response and tumor downstaging in resectable GC. These findings support further investigation of immunotherapy-based perioperative strategies. However, the present evidence is mainly driven by pathological endpoints, and the role of pCR as a surrogate for long-term survival in gastric cancer remains uncertain. Therefore, mature survival data are needed before definitive conclusions regarding clinical practice can be drawn.

## Simple Summary

This systematic review and meta-analysis evaluated whether adding immune checkpoint inhibitors (ICIs) to perioperative chemotherapy improves outcomes in patients with resectable gastric cancer. Six clinical studies including 2890 patients were analyzed. Overall, chemo-immunotherapy significantly improved pathological tumor response, with higher rates of major tumor regression and a marked increase in pathological complete response compared with chemotherapy alone. A trend toward improved lymph node downstaging was also observed, although not statistically significant. Benefits were particularly pronounced in PD-L1-positive tumors and were consistent across age and performance status subgroups. However, R0 resection rates were not significantly different between treatment strategies. Importantly, pathological complete response has not been validated as a surrogate for overall survival in gastric cancer, and whether these pathological gains translate into longer survival remains unknown. These findings suggest that the addition of immunotherapy enhances tumor eradication before surgery but does not clearly increase surgical radicality. Overall, perioperative chemo-immunotherapy appears to be a promising strategy in resectable gastric cancer, although further studies are needed to confirm its impact on long-term survival outcomes.

## 1. Introduction

Gastric cancer (GC) remains a major global health burden and is one of the leading causes of cancer-related mortality worldwide. According to global cancer statistics, gastric cancer ranks among the most frequently diagnosed malignancies and represents the fourth leading cause of cancer-related death, accounting for more than one million new cases and over 760,000 deaths annually [1,2,3]. Despite advances in early detection and multidisciplinary treatment, prognosis remains poor for many patients, particularly due to high rates of recurrence even after curative-intent surgery [3].

For patients with resectable disease, perioperative chemotherapy followed by surgical resection has become the standard of care in Western countries. This approach was initially established by the landmark MAGIC trial, which demonstrated a significant survival benefit for perioperative chemotherapy compared with surgery alone in patients with resectable gastric and gastroesophageal junction cancers [4].

Subsequently, the FLOT4-AIO trial further improved outcomes by introducing the docetaxel-based FLOT regimen, which significantly increased overall survival and pathological response rates compared with previous epirubicin-based regimens [5]. As a result, perioperative FLOT chemotherapy is currently considered the preferred standard regimen for fit patients with resectable gastric cancer in many treatment guidelines.

Nevertheless, despite these therapeutic advances, long-term outcomes remain suboptimal. In this context, the incorporation of immunotherapy into earlier stages of gastric cancer treatment has emerged as a promising strategy.

The neoadjuvant setting may provide a particularly favorable biological context for immunotherapy. Exposure of the immune system to an intact tumor mass during systemic treatment may facilitate antigen presentation and promote a stronger and more durable antitumor immune response [6]. Moreover, histopathological evaluation of tumor regression following neoadjuvant therapy has, therefore, become an important surrogate marker for treatment activity [7,8].

Preclinical and translational studies suggest that chemotherapy may enhance antitumor immunity through several complementary mechanisms, including increased release of tumor-associated antigens, induction of immunogenic cell death, modulation of immune-suppressive cellular populations, and enhancement of T-cell infiltration within the tumor microenvironment. These biological effects provide a strong rationale for combining cytotoxic chemotherapy with immune checkpoint blockade, potentially generating synergistic antitumor activity [8,9]. Pathological complete response (pCR) has been associated with improved survival outcomes in gastric and gastroesophageal junction cancers following neoadjuvant treatment, supporting its potential role as an early marker of treatment efficacy, although its validity as a surrogate endpoint for overall survival remains debated. In addition to pCR, other pathological parameters such as tumor downstaging, nodal response, and tumor regression grading have emerged as clinically relevant indicators of treatment activity. These endpoints may provide valuable information regarding the biological effectiveness of novel perioperative strategies before mature survival data become available [10,11].

Based on this rationale, several recent clinical trials have explored the integration of ICIs with perioperative chemotherapy in resectable gastric and gastroesophageal junction cancers. Early results from studies such as the DANTE trial have suggested that the addition of atezolizumab to perioperative FLOT chemotherapy may enhance tumor regression and increase pathological response rates without compromising surgical feasibility [12]. Other ongoing phase II and III trials are currently investigating similar strategies using different PD-1 or PD-L1 inhibitors in combination with standard perioperative chemotherapy regimens [13,14,15,16].

Similarly, the phase III MATTERHORN trial demonstrated that the addition of durvalumab to perioperative FLOT chemotherapy improved pathological complete response rates, event-free survival (EFS), and overall survival (OS), further supporting the integration of immune checkpoint inhibitors in the perioperative management of resectable disease [17,18].

Although these studies have reported encouraging results, the magnitude and consistency of the benefit associated with chemo-immunotherapy in the perioperative setting remain to be fully defined. Individual trials often have limited sample sizes or heterogeneous patient populations, and direct comparisons across studies are challenging, given reported outcomes that are sometimes heterogeneous in terms of pathological response, survival endpoints, and treatment-related effects.

In particular, the impact of immune checkpoint inhibitors on clinically relevant pathological endpoints, such as pathological complete response (pCR) and tumor downstaging, has not yet been comprehensively quantified, despite evidence suggesting that these responses may correlate with improved long-term outcomes in gastric cancer.

Furthermore, uncertainty remains regarding the patient populations most likely to benefit from this strategy. Biomarkers such as PD-L1 expression, microsatellite instability, tumor mutational burden, and other immune-related features have shown predictive potential in advanced disease, but their role in the perioperative setting remains incompletely characterized [19]. A comprehensive synthesis of the currently available evidence is therefore necessary to better define the therapeutic value of perioperative chemo-immunotherapy and identify factors associated with treatment response.

Therefore, we conducted a systematic review and meta-analysis of phase II–III clinical trials evaluating the addition of ICIs to perioperative chemotherapy in patients with resectable gastric cancer. The aim of this study was to evaluate the efficacy of the integration of immunotherapy with chemotherapy in terms of pathological complete response (pCR) and tumor downstaging, as well as to identify the subgroups of patients who derive the greatest benefit from this combined strategy.

## 2. Materials and Methods

We conducted a systematic review and meta-analysis of phase II–III clinical trials and comparative real-world studies to evaluate the efficacy and safety of ICIs added to perioperative chemotherapy compared with chemotherapy alone in patients with resectable gastric cancer (PROSPERO registration: CRD420261339291).

A systematic literature search was performed in PubMed, Scopus, the Cochrane Library, and major oncology conference proceedings (ASCO and ESMO) from database inception to February 2026. The search strategy was developed using a combination of controlled vocabulary (MeSH terms) and free-text keywords based on the following key concepts: gastric cancer, perioperative treatment, immune checkpoint inhibitors, and chemotherapy.

The PubMed search string was as follows: (“gastric cancer” [Title/Abstract] OR “stomach neoplasms” [Title/Abstract] OR “gastroesophageal junction” [Title/Abstract]) AND (“neoadjuvant” [Title/Abstract] OR “perioperative” [Title/Abstract]) AND (“PD-1” [Title/Abstract] OR “PD-L1” [Title/Abstract] OR “immune checkpoint inhibitor*” [Title/Abstract] OR nivolumab OR pembrolizumab OR durvalumab OR tislelizumab) AND (“chemotherapy” [Title/Abstract]).

**Figure 1:**
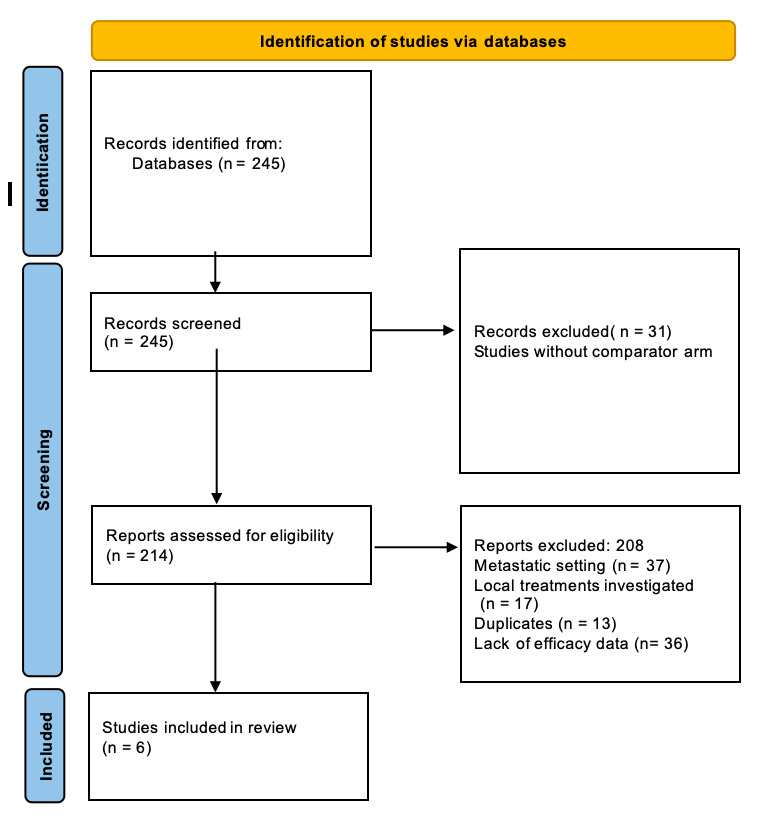
PRISMA flow diagram illustrating the selection process of studies included in the systematic review and meta-analysis.

Two independent reviewers screened titles and abstracts, followed by full-text assessment of potentially eligible studies. Discrepancies were resolved by consensus or consultation with a third reviewer. Review articles, case reports, and single-arm studies were excluded. Additional exclusion criteria included studies evaluating regimens incorporating radiotherapy or other local treatments, as well as trials conducted exclusively in molecularly selected subpopulations (e.g., MSI-H/dMMR tumors). Eligible studies included phase II–III trials and real-world studies comparing perioperative chemotherapy plus ICIs versus chemotherapy alone in resectable gastric cancer with available outcome data. The study was conducted in accordance with PRISMA 2020 guidelines (Figure 1).

Data extraction was independently performed by two investigators. Extracted variables included study design, patient characteristics, treatment regimens, and reported outcomes. The primary endpoint was pathological complete response (pCR), while secondary endpoints included R0 resection rate and nodal downstaging.

The methodological quality of randomized trials was assessed using the Cochrane Risk of Bias 2 (RoB 2) tool. Each domain of the RoB 2 tool (randomization process, deviations from intended interventions, missing outcome data, measurement of the outcome, and selection of the reported result) was assessed (Supplementary Figure S1).

For comparative real-world studies, methodological quality was assessed using the ROBINS-I tool for non-randomized studies of interventions, evaluating bias due to confounding, selection of participants, classification of interventions, missing data, measurement of outcomes, and selection of reported results (Supplementary Figure S2).

Pooled odds ratios (ORs) with 95% confidence intervals (CIs) were calculated using a random-effects model. Statistical heterogeneity was assessed using the I^2^ statistic, with values > 50% indicating substantial heterogeneity. Sensitivity analyses were performed by restricting the analysis to phase III randomized controlled trials to evaluate the robustness of the primary endpoint. Given the limited number of studies (*n* = 6, including only 2 real-world studies), a separate pooled analysis stratified by study design was not performed for secondary endpoints, as it would not provide statistically meaningful estimates. The sensitivity analysis restricted to phase III RCTs was therefore focused on the primary endpoint (pCR), which represents the most clinically relevant outcome of this meta-analysis. All secondary and subgroup analyses should accordingly be regarded as exploratory, given the inclusion of both randomized and real-world evidence.

Publication bias was evaluated by visual inspection of funnel plots (Supplementary Figure S3). Given the small number of included studies (*n* = 6), funnel plot-based assessment of publication bias has limited statistical power and should therefore be interpreted cautiously. A leave-one-out sensitivity analysis confirmed the robustness of the pooled effect estimate. The overall odds ratio remained stable across all iterations (OR range: 2.81–3.77), with all estimates remaining statistically significant (*p* < 0.0001). The pooled heterogeneity showed some variation (I^2^ range: 0–50.8%), with complete elimination of heterogeneity after exclusion of the Shitara et al. 2025 study [15], suggesting that this trial contributed to between-study heterogeneity but did not materially influence the overall effect size (Supplementary Figure S4). All statistical analyses were performed using R software (R Foundation for Statistical Computing, version 4.4.2).

A total of six studies, including 2890 patients, were included in the final analysis.

## 3. Results

Six studies, including a total of 2890 patients, met the eligibility criteria and were included in the final meta-analysis. The main characteristics of the included studies are summarized in Table 1.

**Figure 2:**
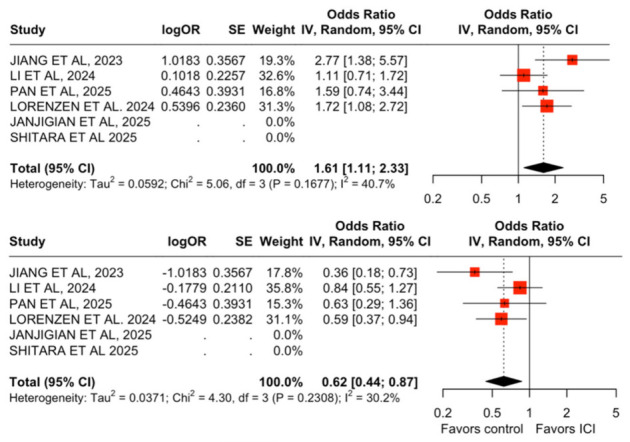
Forest plot of ypT0–T2 tumor response rates (top) and ypT3–T4 residual tumor rates (bottom) for perioperative chemo-immunotherapy versus chemotherapy alone. Data not reported or not available in the original studies are indicated with a dot (·). References: [12,13,14,15,16,18]. Full citation details are provided in Table 1.

The addition of ICIs to perioperative chemotherapy significantly improved tumor regression. Rates of ypT0–T2 response were higher in the chemo-immunotherapy group (OR 1.61, 95% CI 1.11–2.33; *p* = 0.012; I^2^ = 40.7%), whereas ypT3–T4 residual disease was reduced (OR 0.62, 95% CI 0.44–0.87; *p* = 0.0051; I^2^ = 30.2%) (Figure 2).

**Figure 3:**
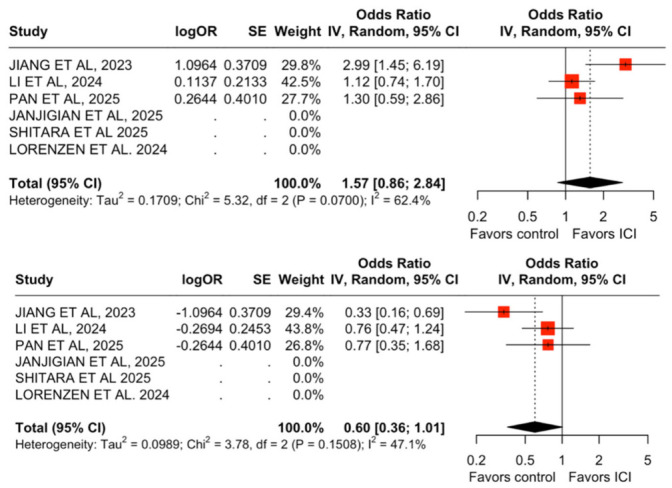
Forest plot of ypN0–1 nodal downstaging rates (top) and ypN2–3 residual nodal disease rates (bottom) for perioperative chemo-immunotherapy versus chemotherapy alone. Data not reported or not available in the original studies are indicated with a dot (·). References: [12,13,14,15,16,18]. Full citation details are provided in Table 1.

Nodal downstaging was also favorably affected. Patients receiving chemo-immunotherapy showed higher rates of ypN0–1 status (OR 1.57, 95% CI 0.86–2.84) and a trend toward reduced ypN2–3 disease (OR 0.60, 95% CI 0.36–1.01); however, these differences did not reach statistical significance (Figure 3).

**Figure 4:**
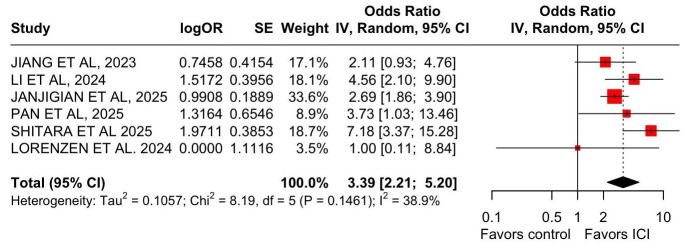
Forest plot of pooled pathological complete response (pCR) for patients treated with perioperative chemo-immunotherapy versus chemotherapy alone. References: [12,13,14,15,16,18]. Full citation details are provided in Table 1.

Absolute pCR rates in the chemo-immunotherapy arms varied across studies, ranging from 13.4% to 26%. Pathological complete response (pCR) rates were markedly increased with chemo-immunotherapy, more than tripling compared with chemotherapy alone (OR 3.39, 95% CI 2.21–5.20; *p* < 0.0001; I^2^ = 38.9%), representing one of the most robust and consistent efficacy signals observed across studies (Figure 4). A sensitivity analysis restricted to phase III randomized trials confirmed the robustness of the primary findings. The addition of ICIs remained significantly associated with a higher pCR rate (OR 3.78, 95% CI 2.14–6.66; *p* < 0.0001), with moderate heterogeneity across studies (I^2^ = 57.4%) (Supplementary Figure S5).

**Figure 5:**
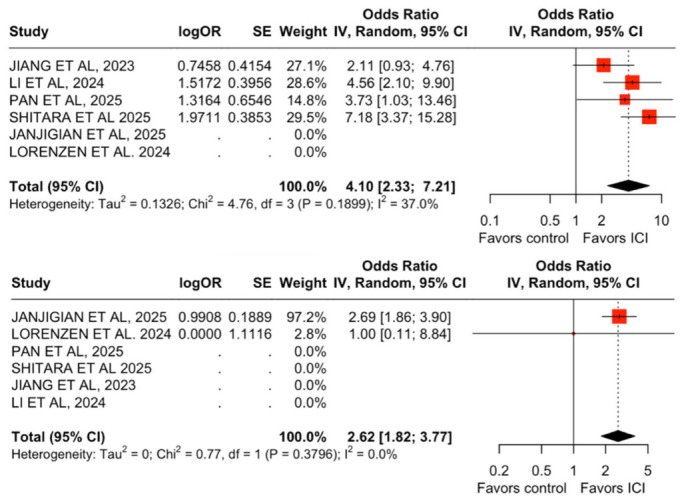
Forest plot of pCR stratified by PD-1 inhibitors (top) and PD-L1 inhibitors (bottom) in perioperative chemo-immunotherapy. Data not reported or not available in the original studies are indicated with a dot (·). References: [12,13,14,15,16,18]. Full citation details are provided in Table 1.

Subgroup analyses demonstrated the greatest benefit with PD-1 inhibitors (OR 4.10, 95% CI 2.33–7.21; *p* < 0.0001; I^2^ = 37%), whereas PD-L1 inhibitors also showed a significant but slightly lower effect (OR 2.62, 95% CI 1.82–3.77; *p* < 0.0001; I^2^ = 0.0%) (Figure 5).

**Figure 6:**
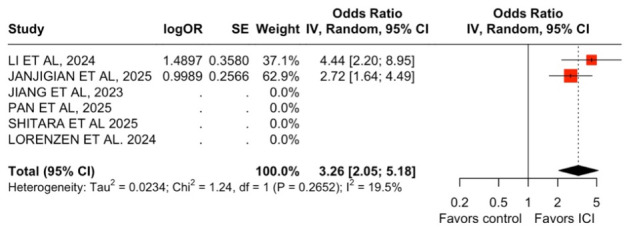
Forest plot of pCR in PD-L1-positive tumors treated with perioperative chemo-immunotherapy versus chemotherapy alone. Data not reported or not available in the original studies are indicated with a dot (·). References: [12,13,14,15,16,18]. Full citation details are provided in Table 1.

The magnitude of benefit was particularly pronounced in PD-L1-positive tumors (CPS/TAP ≥ 1) (OR 3.26, 95% CI 2.05–5.18; *p* < 0.0001; I^2^ = 19.5%) (Figure 6).

**Figure 7:**
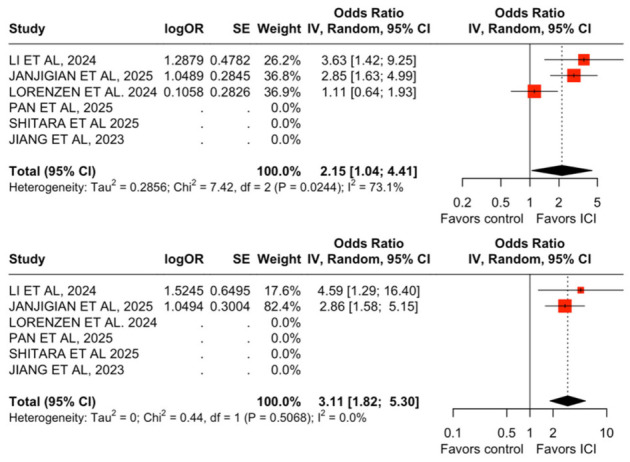
Forest plot of pCR by age subgroup (<65 years vs. ≥65 years) for patients receiving perioperative chemo-immunotherapy. Data not reported or not available in the original studies are indicated with a dot (·). References: [12,13,14,15,16,18]. Full citation details are provided in Table 1.

Treatment benefits were consistent across age and performance status subgroups. In patients aged <65 years, ICIs significantly improved tumor regression (OR 2.15, 95% CI 1.04–4.41; *p* = 0.037; I^2^ = 73.1%), and a comparable magnitude of benefit was observed in those aged ≥65 years (OR 3.11, 95% CI 1.82–5.30; *p* < 0.0001; I^2^ = 0.0%) (Figure 7). Substantial heterogeneity was observed in patients younger than 65 years, which limits the robustness of the pooled estimate. Therefore, the observed treatment effect in this subgroup should be interpreted with caution.

**Figure 8:**
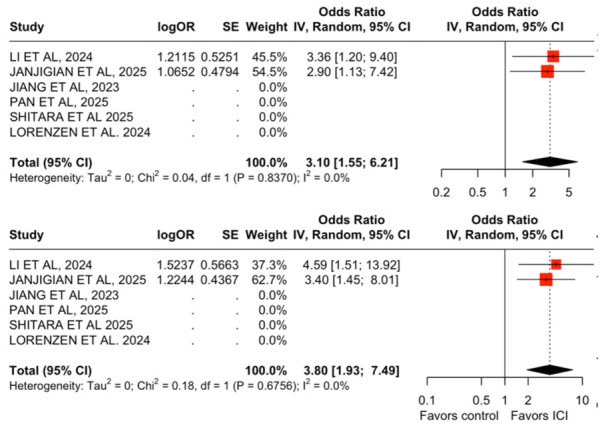
Forest plot of pCR by ECOG performance status (0 vs. 1) in perioperative chemo-immunotherapy. Data not reported or not available in the original studies are indicated with a dot (·). References: [12,13,14,15,16,18]. Full citation details are provided in Table 1.

**Figure 9:**
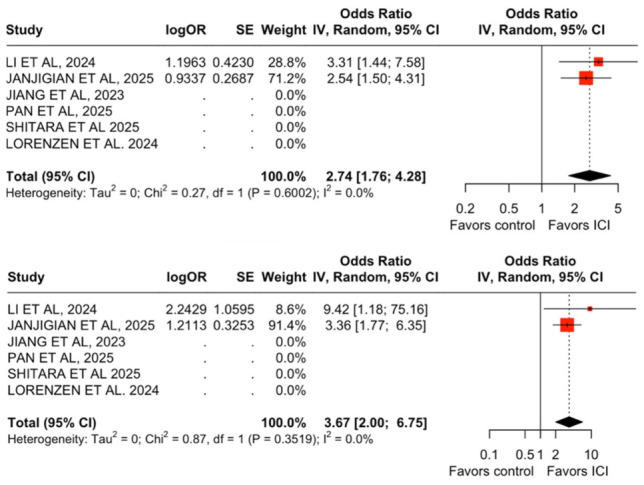
Forest plot of pathological complete response (pCR) for perioperative chemo-immunotherapy versus chemotherapy alone (top: gastric body; bottom: gastroesophageal junction tumors). Data not reported or not available in the original studies are indicated with a dot (·). References: [12,13,14,15,16,18]. Full citation details are provided in Table 1.

Similarly, the addition of ICIs resulted in substantial improvements irrespective of baseline performance status, with significant effects in both ECOG 0 (OR 3.10, 95% CI 1.55–6.21; *p* = 0.0014; I^2^ = 0.0%) and ECOG 1 patients (OR 3.80, 95% CI 1.93–7.49; *p* = 0.0001; I^2^ = 0.0%), supporting the robustness and broad applicability of the treatment effect (Figure 8). Consistent findings were also observed according to tumor site, with a significant benefit of ICIs both in gastric tumors (OR 2.74, 95% CI 1.76–4.28; *p* < 0.0001) and in gastroesophageal junction tumors (OR 3.67, 95% CI 1.99–6.75; *p* < 0.0001), with no evidence of heterogeneity in either subgroup (I^2^ = 0.0%) (Figure 9).

Subgroup analyses were limited by the small number of contributing studies per comparison, which substantially limits statistical robustness. Therefore, these results should be considered hypothesis-generating and interpreted cautiously.

**Figure 10:**
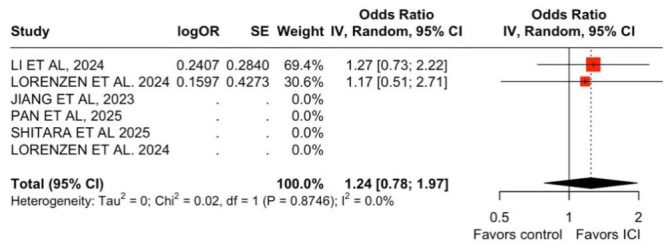
Forest plot of R0 resection rates for perioperative chemo-immunotherapy versus chemotherapy alone. Data not reported or not available in the original studies are indicated with a dot (·). References: [12,13,14,15,16,18]. Full citation details are provided in Table 1.

No statistically significant difference was observed in R0 resection rates between the two treatment groups (OR 1.24, 95% CI 0.78–1.97) (Figure 10).

Safety data were available for a subset of the included studies and were analyzed whenever sufficient information was reported. Overall, the addition of ICIs to perioperative chemotherapy was associated with a statistically significant increase in grade 3–4 adverse events compared with chemotherapy alone (pooled OR 2.11, 95% CI 1.24–3.58; I^2^ = 80.1%; *p* = 0.0001) (Supplementary Figure S6).

Postoperative complications did not differ significantly between treatment groups (pooled OR 1.87, 95% CI 0.54–6.46; *p* = 0.32) (Supplementary Figure S7).

Survival outcomes were reported in two phase III randomized trials (MATTERHORN and KEYNOTE-585). A pooled analysis of event-free survival (EFS) demonstrated a significant benefit with the addition of ICIs to perioperative chemotherapy (pooled HR 0.76, 95% CI 0.66–0.87; *p* < 0.0001), with no evidence of statistical heterogeneity (I^2^ = 0%) (Supplementary Figure S8).

Overall survival (OS) data were also available from the same two studies. The pooled analysis showed a trend toward improved OS with chemo-immunotherapy (pooled HR 0.78, 95% CI 0.61–1.00; *p* = 0.0505), although the result missed conventional statistical significance and moderate heterogeneity was observed (I^2^ = 50.2%) (Supplementary Table S1).

Exploratory univariable meta-regression analyses were performed to investigate potential sources of heterogeneity. No significant association was observed between treatment effect and geographic origin (Asian vs. non-Asian populations), chemotherapy backbone (FLOT vs. fluoropyrimidine-based regimens), PD-L1 status (PD-L1-positive vs. PD-L1-negative tumors), sex distribution, or MSI-H/dMMR status (all *p* > 0.05). Conversely, tumor location significantly influenced treatment effect, with studies including a higher proportion of gastric cancers showing greater pathological benefit compared with those including gastroesophageal junction tumors (*p* = 0.045). Given the limited number of included studies, these findings should be considered exploratory and interpreted with caution.

Immune-related adverse events (irAEs) were reported in only a limited number of studies. Across the available trials, the incidence of irAEs ranged from approximately 4% to 22% in patients receiving chemo-immunotherapy. Because of the limited number of studies and inconsistent reporting, a pooled quantitative analysis was not considered appropriate.

Similarly, permanent treatment discontinuation due to adverse events was inconsistently reported. Among the available studies, discontinuation rates ranged from 9% to 29.9% in the chemo-immunotherapy arms compared with 1% to 22.8% in the chemotherapy-alone groups. Given the limited and heterogeneous reporting of these outcomes, only a descriptive summary is provided.

## 4. Discussion

In this meta-analysis, the addition of immune checkpoint inhibitors (ICIs) to perioperative chemotherapy significantly improved pathological outcomes in patients with resectable gastric cancer. In particular, chemo-immunotherapy was associated with higher rates of tumor regression, with a significant increase in ypT0–T2 responses and a corresponding reduction in residual ypT3–T4 disease. These findings suggest that the incorporation of immunotherapy enhances the depth of tumor response beyond that achieved with chemotherapy alone. Chemotherapy may enhance immunogenic cell death and increase tumor antigen release, potentially synergizing with immune checkpoint blockade, thereby amplifying antitumor immune responses in the neoadjuvant setting [20,21,22].

One of the most clinically relevant findings was the substantial increase in pathological complete response (pCR) rates, which were more than threefold higher in patients treated with chemo-immunotherapy compared with chemotherapy alone. This represents one of the most consistent efficacy signals across the included studies and indicates that perioperative immune checkpoint blockade is associated with improved pathological tumor eradication prior to surgery. Although some studies have suggested an association between pCR and favorable long-term outcomes, the validity of pCR as a surrogate endpoint for overall survival in gastric cancer remains uncertain [23,24]. Pathological endpoints primarily reflect local tumor response and may not fully capture the impact on micrometastatic disease or long-term survival outcomes. Therefore, while the observed improvement in pCR is encouraging, its translation into improved survival outcomes remains to be demonstrated in ongoing studies with longer follow-up.

Notably, pCR rates differed substantially between trials. This discrepancy may reflect differences in chemotherapy backbone (fluoropyrimidine-based regimens versus FLOT), patient selection criteria, or regional distribution of enrolled patients, rather than a true difference in the efficacy of the specific ICI used. These trial-level differences underscore that pooled estimates should be interpreted as an average effect across heterogeneous protocols rather than as evidence of a uniform treatment effect.

Because the primary endpoint (pCR) was specifically validated through a sensitivity analysis restricted to phase III randomized trials, we consider this result to be the most robust finding of the present meta-analysis. In contrast, all secondary and subgroup analyses, which combine randomized and real-world evidence, should be interpreted as exploratory and hypothesis-generating rather than confirmatory.

Our analysis also demonstrated a favorable trend toward nodal downstaging. Patients receiving chemo-immunotherapy showed higher rates of ypN0–1 status and a reduction in ypN2–3 disease, although these differences did not reach statistical significance. The smaller effect observed for nodal downstaging compared with pCR may reflect differences in treatment response between primary tumors and nodal disease; however, this hypothesis cannot be confirmed by the present analysis and warrants further investigation.

This difference may reflect, at least in part, distinct biological and microenvironmental characteristics between primary tumors and lymph nodes, including differences in antigen exposure and immune cell composition, although these remain speculative within the context of the present analysis. Overall, the results suggest a more pronounced effect of chemo-immunotherapy on the primary tumor than on nodal disease [25,26,27,28].

No significant improvement in R0 resection rates was observed with the addition of ICIs, suggesting that the substantial increase in pathological response does not necessarily translate into improved surgical radicality. This finding provides an important counterbalance to the encouraging pCR results and highlights that pathological tumor regression may not fully reflect resectability outcomes in this setting.

Subgroup analyses provided further insight into potential determinants of treatment benefit. The magnitude of pCR improvement appeared greater with PD-1 inhibitors compared with PD-L1 inhibitors, although both classes demonstrated significant activity. These differences should be interpreted with caution because they derive from indirect subgroup comparisons across a limited number of studies rather than from head-to-head randomized comparisons.

Importantly, the benefit of chemo-immunotherapy appeared particularly pronounced in tumors expressing PD-L1. PD-L1 CPS was analyzed as a binary variable according to the definitions adopted in the included studies. Therefore, the potential correlation between increasing CPS/TAP values and treatment benefit could not be explored.

Patients with PD-L1-positive tumors demonstrated significantly higher pCR rates compared with those treated with chemotherapy alone. This observation is consistent with findings in the metastatic setting, where PD-L1 expression has been associated with greater sensitivity to immune checkpoint inhibition [17,29,30,31]. However, PD-L1 remains an imperfect biomarker, with potential variability due to intratumoral heterogeneity and dynamic changes during treatment [19]. Furthermore, the limited availability of biomarker data across the included studies precluded a more comprehensive assessment of its predictive value in the perioperative setting.

The treatment effect was largely consistent across clinically relevant patient characteristics, including ECOG performance status and primary tumor location. Regarding age, a benefit was observed in both patients younger and older than 65 years; however, the estimate in the younger subgroup was associated with substantial heterogeneity (I^2^ = 73.1%), and should therefore be interpreted with more caution.

Gastric cancer (GC) and gastroesophageal junction (GEJ) tumors were analyzed together because they are managed under a unified perioperative strategy in current international guidelines and in the pivotal trials that define the standard of care in this setting, including FLOT4-AIO [5] and MATTERHORN [17,18], both of which enrolled patients with either tumor site under the same treatment protocol. This reflects the anatomical continuity and overlapping histopathological features of the two entities, and supports their combined analysis as a single perioperative population. Nonetheless, GC and GEJ tumors differ biologically in terms of embryological origin, lymphatic drainage patterns, and molecular subtype distribution, which may translate into differential sensitivity to immune checkpoint blockade. The decision to pool these entities in the primary analysis was therefore driven by shared clinical management rather than biological equivalence, and the dedicated subgroup analysis by tumor site was performed specifically to mitigate this limitation. Given that many subgroup analyses were based on a very small number of studies, these findings should be regarded as exploratory and hypothesis-generating rather than definitive, and should be interpreted with appropriate caution.

Although pathological outcomes represented the primary focus of the present meta-analysis, the available survival data provide preliminary evidence regarding the potential clinical impact of perioperative chemo-immunotherapy. A pooled analysis of the available phase III trials showed a significant improvement in event-free survival, whereas the overall survival analysis demonstrated only a borderline statistically significant benefit. However, these findings should be interpreted with considerable caution, as they are based on only two randomized studies and survival data remain relatively immature. Longer follow-up and additional randomized trials are needed to determine whether the improvements in pathological response and event-free survival translate into durable overall survival benefits.

Exploratory meta-regression analyses did not identify any significant association between treatment effect and geographic region (Asian vs. non-Asian populations), chemotherapy backbone (FLOT vs. fluoropyrimidine-based regimens), PD-L1 status, sex distribution, or MSI-H/dMMR status. In contrast, EBV status and tumor mutational burden (TMB) could not be evaluated due to the absence of consistent reporting across the included studies, precluding a comprehensive assessment of these biomarkers. Taken together, the lack of consistent biomarker reporting across studies means that current evidence remains insufficient to reliably identify which patient subgroups derive the greatest benefit from perioperative immunotherapy, and biomarker-driven patient selection should be regarded as an open research question rather than a validated clinical strategy at this stage.

Safety is a key consideration when introducing immunotherapy into the perioperative setting, where treatment-related toxicity may influence treatment completion, surgical timing, and postoperative recovery. In the present analysis, the addition of ICIs was associated with a higher incidence of severe treatment-related adverse events. In contrast, no statistically significant difference was observed in postoperative complications. Likewise, immune-related adverse events and treatment discontinuation could only be described qualitatively because of limited and heterogeneous reporting. Therefore, the overall safety profile of perioperative chemo-immunotherapy cannot be fully characterized based on the currently available evidence.

Several limitations should be acknowledged. First, heterogeneity across the included studies in terms of treatment regimens, patient selection, pathological assessment, and PD-L1 evaluation methods (including different scoring systems and cut-offs, such as CPS and TAP) may influence pooled estimates. Second, some endpoints, particularly nodal downstaging, may have been underpowered. Furthermore, the present analysis was primarily based on pathological outcomes, particularly pCR. Although pCR represents an important indicator of treatment activity, its role as a validated surrogate for overall survival in resectable gastric cancer remains uncertain. Consequently, the clinical implications of the observed pathological improvements should be interpreted with caution until mature survival data become available. Third, the exclusion of studies incorporating radiotherapy, other local therapies, or exclusively molecularly selected populations (such as MSI-H/dMMR tumors) may increase internal consistency but limit external generalizability. Additionally, the inclusion of both randomized trials and real-world studies may introduce structural heterogeneity and potential selection bias. The DRAGON IV trial was included because it represents one of the few available phase III comparative studies evaluating a PD-1-based perioperative strategy against standard chemotherapy, thereby contributing important high-level evidence to the meta-analysis [14]. However, it is acknowledged that this study also incorporated an antiangiogenic agent (rivoceranib) in the experimental arm, which differentiates its treatment backbone from the other included trials. This introduces an additional source of clinical heterogeneity.

Finally, several subgroup and survival analyses were based on only a limited number of studies and should therefore be regarded as exploratory.

Despite these limitations, this study provides a comprehensive synthesis of the current evidence supporting perioperative chemo-immunotherapy in resectable gastric cancer. The consistent improvements in tumor regression and pCR across multiple studies suggest that the addition of immunotherapy to perioperative chemotherapy is associated with enhanced pathological response in resectable gastric cancer. However, these results should be interpreted in the context of immature survival data and the uncertain role of pathological endpoints as surrogates for long-term clinical benefit.

Taken together, these findings suggest that integrating immune checkpoint inhibitors into perioperative chemotherapy is a promising strategy for improving pathological outcomes in resectable gastric cancer. Whether these benefits translate into improved survival and justify changes in clinical practice remains to be determined.

## 5. Conclusions

In conclusion, this analysis provides an encouraging perspective on the potential of perioperative chemo-immunotherapy in gastric cancer. Overall, the addition of ICIs to perioperative chemotherapy substantially increases pathological response and tumor downstaging in resectable gastric cancer. While the consistent improvements in pathological outcomes are a promising sign of progress, they represent an initial step in our understanding. Importantly, although pCR is an attractive early endpoint, its role as a validated surrogate for overall survival in resectable gastric cancer remains uncertain. Therefore, pathological improvements should not be interpreted as definitive evidence of long-term clinical benefit until mature survival data become available. Similarly, biomarker-driven selection should be regarded as a research priority rather than a validated clinical strategy at this stage.

Future studies will be pivotal in clarifying the long-term clinical significance of these responses and in identifying which patient populations may derive the most meaningful benefit from this emerging therapeutic approach.

## Figures and Tables

**Table 1:** Main characteristics of phase II–III clinical trials evaluating perioperative chemotherapy with or without immune checkpoint inhibitors (ICIs) in resectable gastric cancer.

Author, Year	Trial	Phase (N, Experimental Arm)	Experimental Treatment	pCR (%)	Tumor Stage	PD-L1 Assessment (% Positive)	Geographic Region (%)	Median Follow-up
JIANG et al., 2023 [13]	—	Real-world (50)	Tislelizumab + SOX/FOLFOX	26	cT2-T3a N any Or N+ EGJ or gastric	NA	Asian (100)	—
LI et al., 2024 [14]	DRAGON IV	III (180)	Camrelizumab + Rivoceranib + SOX	18.3	T3-4aN+ EGJ or gastric	CPS (78)	Asian (100)	—
JANJIGIAN et al., 2025 [18]	MATTERHORN	III (474)	Durvalumab + FLOT	19.2	stage II through IVa EGJ or gastric	TAP (89.9)	White (67.7) Asian (20.3) Black (1.3)	31.5 mos
PAN et al., 2025 [16]	—	Real-world single center (49)	ICIs + SOX/CAPOX/nab paclitaxel+S-1	22.4	cT2-T3a N any Or N+ EGJ or gastric	CPS (79.6)	Asian (100)	30 mos
SHITARA et al., 2025 [15]	KEYNOTE-585	III (502)	Pembrolizumab + FLOT/XP/FP	12.9	≥T3 or N+ EGJ or gastric	CPS (73)	Asian (47) White 27)	47.7 mos
LORENZEN et al., 2024 [12]	DANTE	II/III (146)	Atezolizumab + FLOT	24	≥cT2 and/or cN+EGJ or gastric	CPS (57.6)	White (100)	—

## Data Availability

All data supporting the findings of this study were extracted from previously published clinical trials and publicly available sources. No original individual patient data were generated or analyzed in this study. Further details can be obtained from the corresponding author upon reasonable request.

## Supplementary Materials

The following supporting information can be downloaded at https://www.mdpi.com/article/10.3390/cancers18142270/s1: Figure S1. Risk of bias assessment of the included randomized controlled trials. Li et al., 2024 [14]; Janjigian et al., 2025 [18]; Shitara et al., 2025 [15]. Figure S2. Risk of bias assessment of the included comparative real-world studies using the ROB-INS-I tool for non-randomized studies of interventions. Jiang et al., 2023 [13]; Pan et al., 2025 [16]. Figure S3. Funnel plot assessing publication bias for the pooled analysis of pathological complete response in patients receiving perioperative chemo-immunotherapy. Figure S4. Leave-one-out sensitivity analysis of the pooled odds ratio. Jiang et al., 2023 [13]; Li et al., 2024 [14]; Janjigian et al., 2025 [18]; Pan et al., 2025 [16]; Shitara et al., 2025 [15]; Lorenzen et al., 2024 [12]. Figure S5. Forest plot of the sensitivity analysis restricted to phase III randomized trials for pathological complete response (pCR). Li et al., 2024 [14]; Janjigian et al., 2025 [18]; Shitara et al., 2025 [15]; Lorenzen et al., 2024 [12]. Figure S6. Forest plot of grade 3–4 treatment-related adverse events comparing perioperative chemo-immunotherapy versus chemotherapy alone in patients with resectable gastric cancer. Jiang et al., 2023 [13]; Li et al., 2024 [14]; Janjigian et al., 2025 [18]; Pan et al., 2025 [16]; Shitara et al., 2025 [15]; Lorenzen et al., 2024 [12]. Figure S7. Forest plot of postoperative complications comparing perioperative chemo-immunotherapy versus chemotherapy alone in patients with resectable gastric cancer. Jiang et al., 2023 [13]; Li et al., 2024 [14]; Janjigian et al., 2025 [18]; Pan et al., 2025 [16]; Shitara et al., 2025 [15]. Figure S8. Forest plots of pooled event-free survival (EFS) (top) and overall survival (OS) (bottom) comparing perioperative chemo-immunotherapy versus chemotherapy alone in patients with resectable gastric cancer. Jiang et al., 2023 [13]; Li et al., 2024 [14]; Janjigian et al., 2025 [18]; Pan et al., 2025 [16]; Shitara et al., 2025 [15]; Lorenzen et al., 2024 [12]. Table S1. Methodological workflow and validation checklist for the Prisma framework.

## Abbreviations

The following abbreviations are used in this manuscript:

CI	Confidence Interval
CPS	Combined Positive Score
dMMR	Deficient Mismatch Repair
ECOG PS	Eastern Cooperative Oncology Group Performance Status
EFS	Event-Free Survival
GC	Gastric Cancer
ICIs	Immune Checkpoint Inhibitors
OR	Odds Ratio
OS	Overall Survival
pCR	Pathological Complete Response
PD-1	Programmed Cell Death Protein 1
PD-L1	Programmed Death-Ligand 1
PRISMA	Preferred Reporting Items for Systematic Reviews and Meta-Analyses
ROB 2	Risk of Bias 2 tool
TAP	Tumor Area Positivity
TPS	Tumor Proportion Score
ypN	Pathological Nodal Stage (post-neoadjuvant)
ypT	Pathological Tumor Stage (post-neoadjuvant)
ASCO	American Society of Clinical Oncology
ESMO	European Society for Medical Oncology
