# Supplementary material for: Impact of Adding Immune Checkpoint Inhibitors to Neoadjuvant Chemotherapy on pCR and Tumor Downstaging in Resectable Gastric Cancer: A Meta-Analysis
Source: Cancers (Basel). 2026 Jul 15;18(14):2270. doi: 10.3390/cancers18142270 (PMC13406764; doi:10.3390/cancers18142270)
Supplement: Supplementary file 1 [file cancers-18-02270-s001.zip › cancers-4378703-supplementary.pdf]

cancers

Supplementary Materials

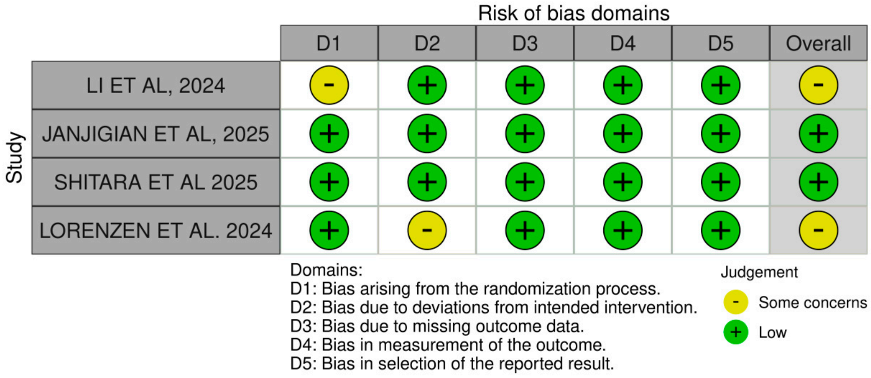

Figure S1. Risk of bias assessment of the included randomized controlled trials. Li et al,. 2024[14]; Janjigian et al,. 2025 [18];Shitara et al,. 2025 [15]

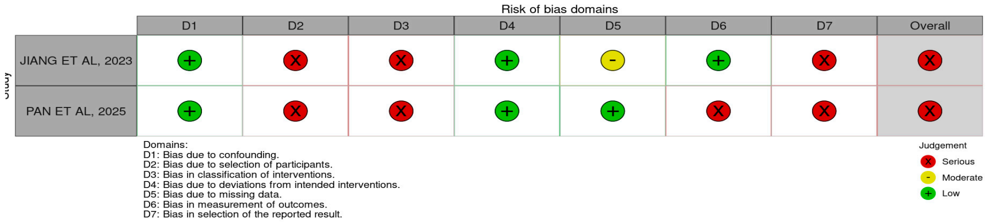

Figure S2. Risk of bias assessment of the included comparative real-world studies using the ROBINS-I tool for non-randomized studies of interventions. Jiang et al,. 2023 [13]; Pan et al,. 2025 [16]

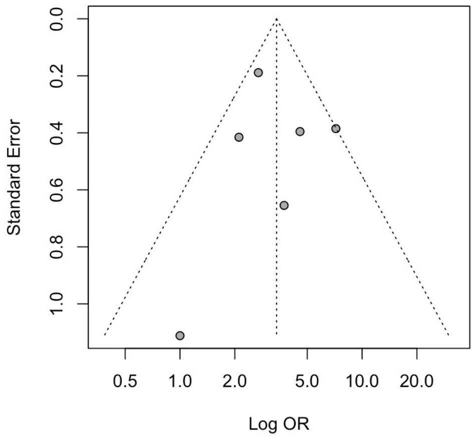

Figure S3. Funnel plot assessing publication bias for the pooled analysis of pathological complete response in patients receiving perioperative chemo-immunotherapy.

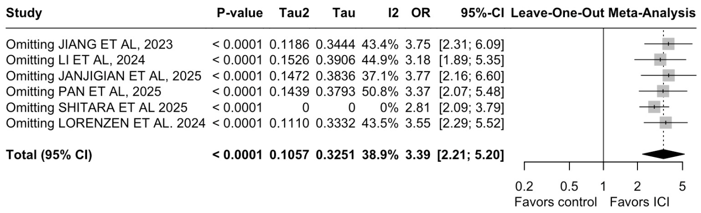

Figure S4. Leave-one-out sensitivity analysis of the pooled odds ratio. Jiang et al,. 2023 [13]; Li et al,. 2024[14]; Janjigian et al,. 2025 [18]; Pan et al,. 2025 [16];Shitara et al,. 2025 [15]; Lorenzen et al,. 2024 [12]

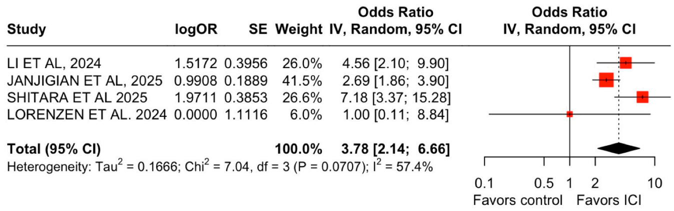

Figure S5. Forest plot of the sensitivity analysis restricted to phase III randomized trials for pathological complete response (pCR). Li et al,. 2024[14]; Janjigian et al,. 2025 [18]; Shitara et al,. 2025 [15]; Lorenzen et al,. 2024 [12]

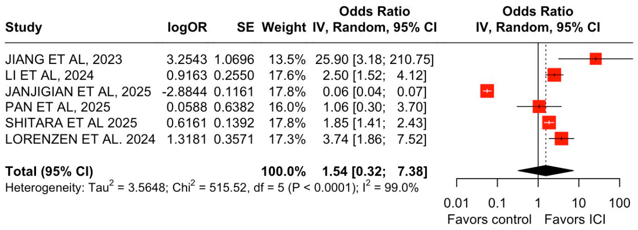

Figure S6. Forest plot of grade 3–4 treatment-related adverse events comparing perioperative chemo-immunotherapy versus chemotherapy alone in patients with resectable gastric cancer. Jiang et al,. 2023 [13]; Li et al,. 2024[14]; Janjigian et al,. 2025 [18]; Pan et al,. 2025 [16];Shitara et al,. 2025 [15]; Lorenzen et al,. 2024 [12]

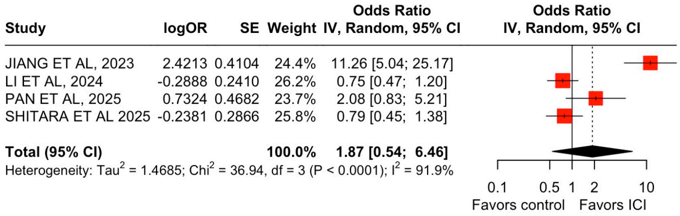

Figure S7. Forest plot of postoperative complications comparing perioperative chemo-immunotherapy versus chemotherapy alone in patients with resectable gastric cancer. Jiang et al,. 2023 [13]; Li et al,. 2024[14]; Janjigian et al,. 2025 [18]; Pan et al,. 2025 [16];Shitara et al,. 2025 [15]

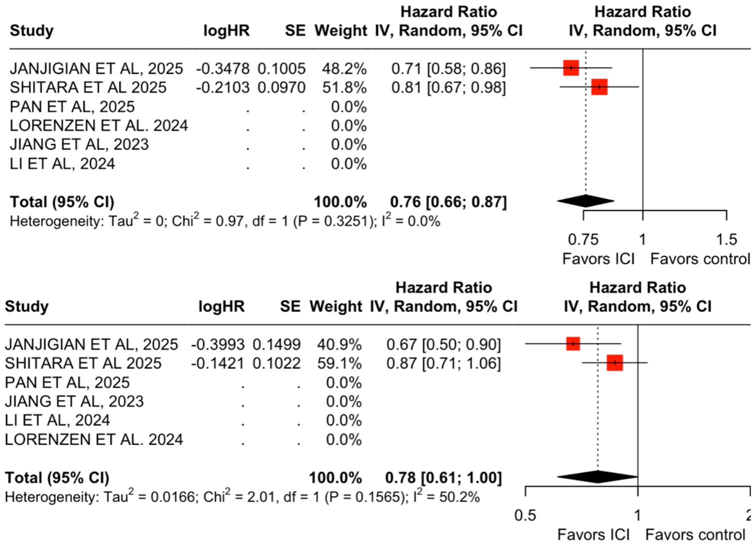

Figure S8. Forest plots of pooled event-free survival (EFS) (top) and overall survival (OS) (bottom) comparing perioperative chemo-immunotherapy versus chemotherapy alone in patients with resectable gastric cancer. Jiang et al,. 2023 [13]; Li et al,. 2024[14]; Janjigian et al,. 2025 [18]; Pan et al,. 2025 [16];Shitara et al,. 2025 [15]; Lorenzen et al,. 2024 [12]

Table S1. Methodological workflow and validation checklist for the Prisma framework.

Section/Topic	Item	Checklist Item	Reported on Page
Title	1	Identify the report as a systematic review and/or meta-analysis.	1
**Section/Topic**	**Item**	**Checklist Item**	**Reported on Page**
Abstract	2	See PRISMA 2020 for Abstracts checklist (structured summary including background, methods, results, conclusions, registration).	8
**Section/Topic**	**Item**	**Checklist Item**	**Reported on Page**

Section/Topic	Item	Checklist Item	Reported on Page
Rationale	3	Describe the rationale for the review in the context of existing knowledge.	3
Objectives	4	Provide an explicit statement of the objective(s) or question(s) the review addresses.	4
**Section/Topic**	**Item**	**Checklist Item**	**Reported on Page**
Eligibility criteria	5	Specify inclusion and exclusion criteria for the review and how studies were grouped for syntheses.	4
Information sources	6	Specify all databases, registers, websites, organizations, reference lists searched and date last searched.	4
Search strategy	7	Present the full search strategies for all databases, including filters and limits used.	4
Selection process	8	Specify methods used to decide whether a study met inclusion criteria, including number of reviewers and independence.	4
Data collection process	9	Specify methods used to collect data from reports, including number of reviewers and process for resolving disagreements.	4
Data items	10a	List and define all outcomes for which data were sought (e.g., DFS, OS).	4
	10b	List and define other variables sought (e.g., tumor type, stage, ICI class, follow-up).	4
Risk of bias assessment	11	Specify methods used to assess risk of bias (e.g., ROB2), including number of reviewers.	4
Effect measures	12	Specify effect measures used (e.g., hazard ratios with 95% CI).	4
Synthesis methods	13a	Describe process for deciding which studies were eligible for each synthesis.	4
	13b	Describe methods to prepare data for synthesis.	4
	13c	Describe methods used to synthesize results (e.g., random-effects model).	4
	13d	Describe methods used to explore heterogeneity (e.g., I^2, meta-regression).	4
	13e	Describe sensitivity analyses, if performed.	4
Reporting bias assessment	14	Describe methods used to assess risk of bias due to missing results (e.g., funnel plot, Egger test).	4
Certainty assessment	15	Describe any methods used to assess certainty of evidence (e.g., GRADE), if applicable.	4
**Section/Topic**	**Item**	**Checklist Item**	**Reported on Page**
Study selection	16a	Describe results of search and selection process (include PRISMA flow diagram).	4
	16b	Cite studies excluded after full-text review with reasons.	4
Study characteristics	17	Cite each included study and present characteristics.	4
Risk of bias in studies	18	Present assessments of risk of bias for each included study.	4
Results of individual studies	19	Present summary statistics and effect estimates for each study.	4
Results of syntheses	20a	Summarize characteristics and risk of bias among contributing studies.	5
	20b	Present results of statistical syntheses (HR, 95% CI, I^2).	5
	20c	Present results of investigations of heterogeneity (e.g., meta-regression).	5
	20d	Present results of sensitivity analyses, if conducted.	5

Section/Topic	Item	Checklist Item	Reported on Page
Reporting biases	21	Present assessments of reporting bias (e.g., funnel plot results, Egger test).	5
Certainty of evidence	22	Present assessments of certainty in evidence, if performed.	5
**Section/Topic**	**Item**	**Checklist Item**	**Reported on Page**
Discussion	23a	Provide a general interpretation of results in context of other evidence.	9-10
	23b	Discuss limitations of the included evidence.	9-10
	23c	Discuss limitations of the review process.	9-10
	23d	Discuss implications for practice, policy, and future research.	9-10
**Section/Topic**	**Item**	**Checklist Item**	**Reported on Page**
Discussion	23a	Provide a general interpretation of results in context of other evidence.	9-10
	23b	Discuss limitations of the included evidence.	9-10
	23c	Discuss limitations of the review process.	9-10
	23d	Discuss implications for practice, policy, and future research.	9-10
**Section/Topic**	**Item**	**Checklist Item**	**Reported on Page**
Registration and protocol	24a	Provide registration information (e.g., PROSPERO number).	4
	24b	Indicate where the protocol can be accessed.	4
	24c	Describe and explain any amendments to protocol.	4
Support	25	Describe sources of financial or non-financial support.	12
Competing interests	26	Declare competing interests.	12
Availability of data/materials	27	Report which materials are publicly available and where.	12
